# Ultrasound diagnosis of transverse sinus hypoplasia using flow profiles of the internal jugular vein

**DOI:** 10.1371/journal.pone.0181119

**Published:** 2017-07-13

**Authors:** A-Ching Chao, Ke Han, Feng-Chi Chang, Hung-Yi Hsu, Chih-Ping Chung, Wen-Yung Sheng, Lung Chan, Jiang Wu, Han-Hwa Hu

**Affiliations:** 1 Department of Neurology, College of Medicine, Kaohsiung Medical University, Kaohsiung, Taiwan; 2 Department of Neurology, Kaohsiung Medical University Hospital, Kaohsiung, Taiwan; 3 Department of Neurology and Neuroscience Center, First Hospital of Jilin University, Changchun, Jilin, China; 4 Department of Radiology, Taipei Veterans General Hospital and National Yang Ming University, Taipei, Taiwan; 5 Department of Neurology, Tungs’ Taichung Metro Harbor Hospital, Taichung, Taiwan; 6 Department of Neurology, Taipei Veterans General Hospital and National Yang-Ming University, Taipei, Taiwan; 7 Department of Neurology, Taipei Medical University-Shaung Ho Hospital, Taipei, Taiwan; 8 Cerebrovascular Treatment and Research Center, College of Medicine, Taipei Medical University, Taipei, Taiwan; National Natural Science Foundation of China, CHINA

## Abstract

Accurate diagnosis of subtypes of transverse sinus (TS) hypoplasia requires more expensive methods like magnetic resonance (MR) imaging. We hypothesized ultrasound findings of the internal jugular vein (IJV) can be surrogate indicators for diagnosis of TS hypoplasia. MR images were reviewed in 131 subjects to evaluate TS diameter and the location and degree of venous flow stenosis and obstruction. Ultrasound parameters including the cross-sectional lumen area (CSA), time-average-mean velocity (TAMV), and flow volume (FV) at each IJV segment were also evaluated. Sixty-nine subjects had TS hypoplasia based on MRV criteria, of which 39 TS hypoplasia were considered a subtype of TS hypoplasia, which is secondary to the downstream venous compression/stenosis or left brachiocephalic vein. In the ultrasound study, the CSA of the IJV ipsilateral to TS hypoplasia was significantly smaller. Further, a contralateral/ipsilateral IJV CSA ratio >1.55 provided good sensitivity, specificity, and positive predictive value for discriminating TS hypoplasia.

## Introduction

Asymmetry of the transverse sinus (TS) is a common finding with unilateral hypoplasia or aplasia in 20–39% of cases, and was previously considered to be a normal anatomical variation [1–3]. However, increasing evidence suggests that asymmetry of the TS is associated with the manifestation and progression of a number of neurological disorders [4–7]. Adverse neurological effects that have been associated with TS hypoplasia include: a prolonged cerebral circulation time and impaired cerebral autoregulation linked to postcarotid-stenting hyperperfusion syndrome [4], severe brain edema in middle cerebral artery infarction as a result of increased venous outflow resistance [5], high-altitude headache [6], and white matter hyperintensities in patients with Parkinson’s disease [7]. Thus, TS hypoplasia is increasingly considered to be an abnormality that significantly affects cerebral hemodynamic regulation.

Recently, asymmetry of the TS was successfully measured using magnetic resonance venography (MRV) and contrast T1-weighted magnetic resonance imaging (contrast T1) in transient global amnesia (TGA) and transient monocular blindness (TMB) [8,9]. Furthermore, two types of TS hypoplasia (anatomical TS hypoplasia and flow-related TS hypoplasia) were recently distinguished using a post-hoc analysis of the discrepancy and conformance of TS diameters measured by MRV and contrast T1 [10]. While anatomical TS hypoplasia is associated with TS diameter conformance, flow-related TS hypoplasia only appears on MRV due to downstream internal jugular vein (IJV) or brachiocephalic vein (BCV) compression/stenosis, and has been associated with neurological disorders of venous outflow impairment [8–10]. However, previous TS hypoplasia studies reporting adverse neurological effects included both the anatomical TS hypoplasia and flow-related TS hypoplasia and did not pay attention to the outflow impairment [4–7]. Given that different types of TS hypoplasia may have different clinical implications, the use of MR imaging modalities (including MRV and contrast T1) to distinguish the type of TS hypoplasia is necessary. Moreover, a simple non-invasive test of physiological flow variables is necessary to replace more expensive MR imaging studies with contrast or invasive angiography for TS hypoplasia visualization and diagnosis in routine clinical practice.

Anatomically, the IJVs provide direct venous drainage from the TSs. One autopsy study revealed that TS diameter correlates with the size of the IJV [11]. Imaging duplex ultrasound is a simple and non-invasive test that has been clinically used to evaluate the morphology of IJV, and the resting flow profiles and hemodynamic changes in the IJV during phases of respiration. Of note, ultrasound has the advantage to study the dynamic hemodynamic changes of the IJV during respiration. Therefore, we hypothesized that imaging duplex ultrasound of the IJV might be useful for predicting the existence of TS hypoplasia and distinguishing the subtype type of TS hypoplasia in patients. In previous studies, we have routinely used ultrasound in tandem with MR imaging to study venous abnormalities in TGA, TMB, and panic disorder [8,9,12]. Thus, we conducted an analysis of these patient populations to evaluate correlations between ultrasound findings related to the flow volume (FV) and lumen of the IJVs and MR imaging findings of TS hypoplasia. Our data suggest that ultrasound of the IJVs is useful as a complementary or surrogate diagnostic tool for TS hypoplasia.

## Methods

### Subjects

The study protocols of TGA, TMB and Panic disorders for the study were approved by the Ethics Committee of Taipei Veterans General Hospital in compliance with the Declaration of Helsinki and all participants provided informed written consent with their signatures. The consent from the parents or guardians of the minors included in these studies were also obtained. A total of 131 subjects who were previously enrolled in prospective studies of venous outflow impairment in TGA, TMB, and panic disorder were included [8,9,12], which comprised 45 TGA, 24 TMB, 21 panic disorder and 41 control subjects with complete MRI, MRV and ultrasound examinations.

TGA patients were recruited consecutively from April 14, 2006 to May 28, 2007, from the Neurology Department of Taipei Veterans General Hospital. All TGA patients were examined by the same neurologist and TGA was diagnosed according to the criteria modified and validated by Hodges and Warlow [13]. Age- and gender-matched subjects for the control group were recruited from individuals receiving physical check-ups and had no history of neurological symptoms [8].

TMB patients were recruited consecutively from February 16, 2005 to February 15, 2006, as outpatients from the Neurology Department of Taipei Veterans General Hospital, and from referrals for cerebrovascular survey by ophthalmologists or other physicians. All patients were examined by the same neurologist and were questioned about the characteristics of their transient vision loss using a standardized questionnaire. Age- and gender-matched subjects for the control group were recruited from individuals receiving physical check-ups and had no history of carotid stenosis or previous visual problems [9].

Panic disorder patients, which has been hypothesized because many panic patients have mitral valve prolapse, and we would like to know if they also have valve problem of IJV, or could be a disorder associated with venous outflow impairment [12], were recruited consecutively as referrals from the psychological clinic at Taipei Veterans General Hospital (Dr. Hong) from June 8, 2006 to June 7, 2007, and were diagnosed according to the Diagnostic and Statistical Manual of Mental Disorders, 4th ed. (DSM-IV-TR) criteria [14]. Patients who were unwilling to participate or had a history of hypertension, diabetes, smoking, stroke, ischemic heart disease, congestive heart disease, arrhythmia, pulmonary diseases, carotid or intracranial artery stenosis, and malignancies were excluded [12].

In total, 131 patients and controls that had complete studies with MR imaging and ultrasound were included.

### MR imaging

Subjects were imaged using a 1.5T MR imaging scanner (GE Medical Systems, Milwaukee, WI, USA). The imaging protocol included time-resolved imaging of contrast kinetics (TRICKS), contrast-enhanced axial T1-weighted magnetic resonance imaging (contrast T1), and MRV. MRV angiography was phase contrast-based (contrast-free) and acquired using Inhance 3D Velocity software (GE Medical Systems, Milwaukee, WI, USA). We used the following MR sequences: (1) Inhance 3D Velocity MRV: sagittal plane repetition time (TR)/echo time (TE)/flip angle (FA) = 11.7/4.5/10°, matrix size = 320 × 256, field of view (FOV) = 24 cm × 21.6 cm, slice thickness = 1.4 mm with interpolation to a 0.7 mm slice interval using a parallel imaging technique, acceleration factor = velocity encoding, 25 cm/s; (2) TRICKS: coronal plane TR/TE/FA = 3.1/1.1/30°, matrix size = 320 × 192, FOV = 34 cm × 30.6 cm, slice thickness = 3.2 mm with interpolation to a 0.8 mm slice interval; (3) contrast-enhanced T1 spoiled-gradient recalled (SPGR) acquisition in the steady state MR sequence: axial plane TR/TE/FA = 8.6/2.5/15°, matrix size = 320 × 256, FOV = 24 cm × 18 cm, slice thickness = 3 mm. Included patients and controls had completed all 3 MR imaging studies.

IJV morphology on contrast T1 images was assessed at the upper IJV (at the C1-C2 level), middle IJV (at the C3-C5 level), and lower IJV (at the C6-T2 level). IJV compression/stenosis was evaluated according to the following scale by. Zaharchuk et al.: grade 0 = normal round or ovoid appearance; grade 1 = mild flattening; grade 2 = moderate flattening; grade 3 = severe flattening or not visualized [15].

Left BCV obstruction on TRICKS imaging was graded based on the observation of a filling defect according to the following scale: grade 0 = normal or compression ≤ 20%; grade 1 = compression > 20% and ≤ 80%; grade 2 = compression > 80%; grade 3 = grade 2 plus the presence of different types of venous collaterals [8]. The vertebral/intraspinal/neck collaterals were evaluated with a focus on the posterior condylar veins and collaterals of the vertebral venous system.

TS diameter on MRV and contrast T1 images was measured in cm at the mid-lateral portion of the TS using the method proposed by Fofi et al. [1], as the mid-lateral portion of the TS was easily identified and measured with certainty in almost all cases. TS morphology was graded according to a scale modified from Fofi et al.: grade 0 = TS symmetry or TS asymmetry ≤ 10%; grade 1 = TS asymmetry > 10% and ≤ 50%; grade 2 = TS asymmetry > 50%; grade 3 = aplasia or the absence of TS signal [1]. TS hypoplasia was defined as an indexed TS > 50% of the contralateral TS, including grades 2 and 3 [10].

One neuroradiologist and one neurologist re-examined all MR images. Both physicians were well trained in the interpretation of neuroimaging and were blinded to the clinical characteristics of subjects. A consensus meeting was conducted to discuss any problems or disagreements. The inter-rater reliability coefficient was greater than 0.91 for all assessments.

### Ultrasound

Color-coded duplex sonography was conducted using a 7-MHz linear transducer (iU22; Philips Medical Systems, Andover, MA, USA) by a single technician who was blinded to the clinical characteristics of subjects. The method of ultrasound examination of the extracranial venous system has been reported elsewhere [8,9,12,15–18].

**Fig 1:**
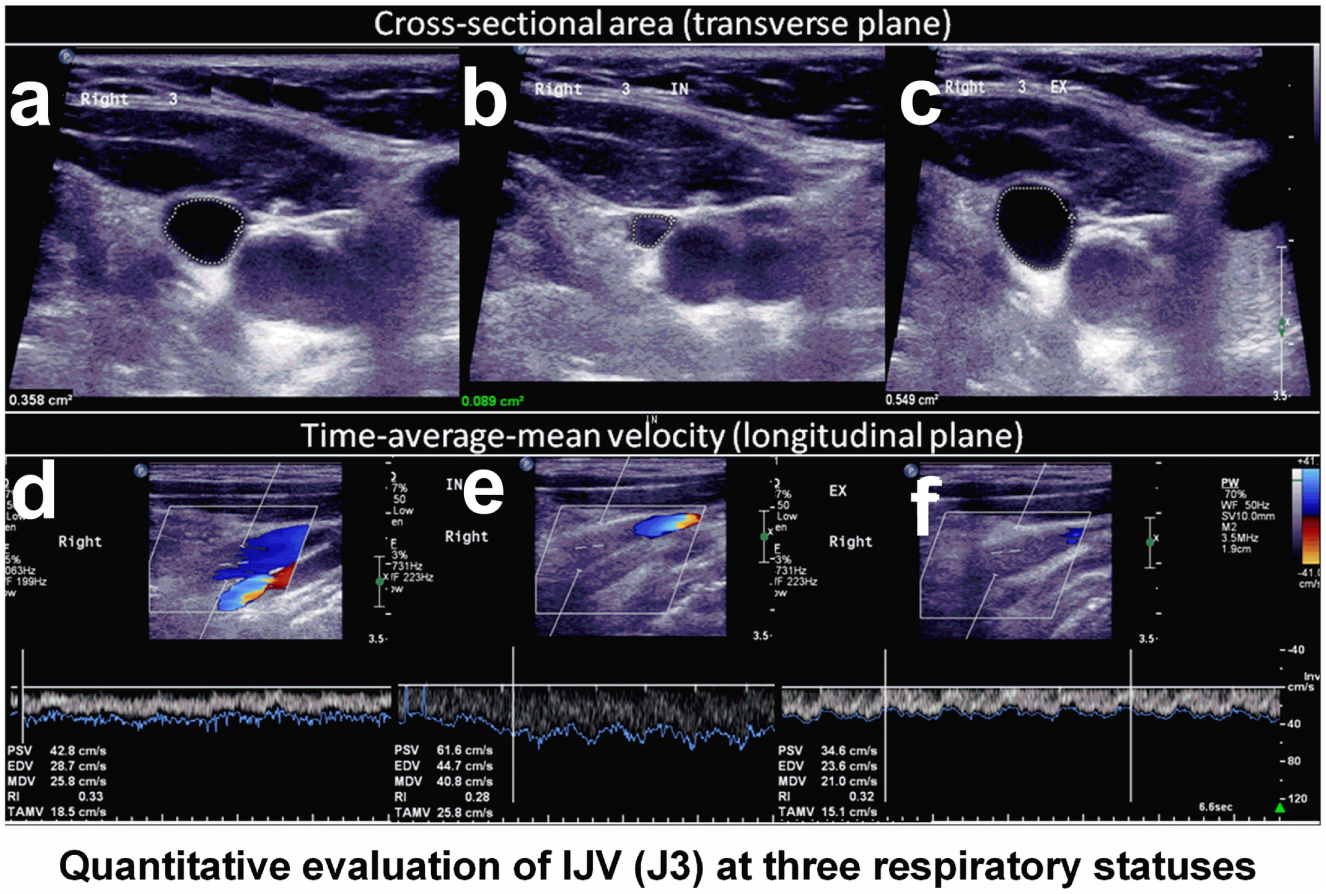
Quantitative evaluation of the CSA and TAMV of the IJV during three respiratory statuses. The cross-sectional lumen areas (CSAs) and time-average-mean velocity (TAMVs) of the upper segment of the internal jugular vein (IJV, J3) were recorded during brief apnea after three respiratory statuses: (A, D) rest (normal respiratory status, (B, E) deep inspiration, and (C, F) expiration. Compared with rest, CSA was decrease (B) while TAMV was increased (E) during deep inspiration, and CSA was increased (C) while TAMV was decreased (F) during expiration.

**Fig 2:**
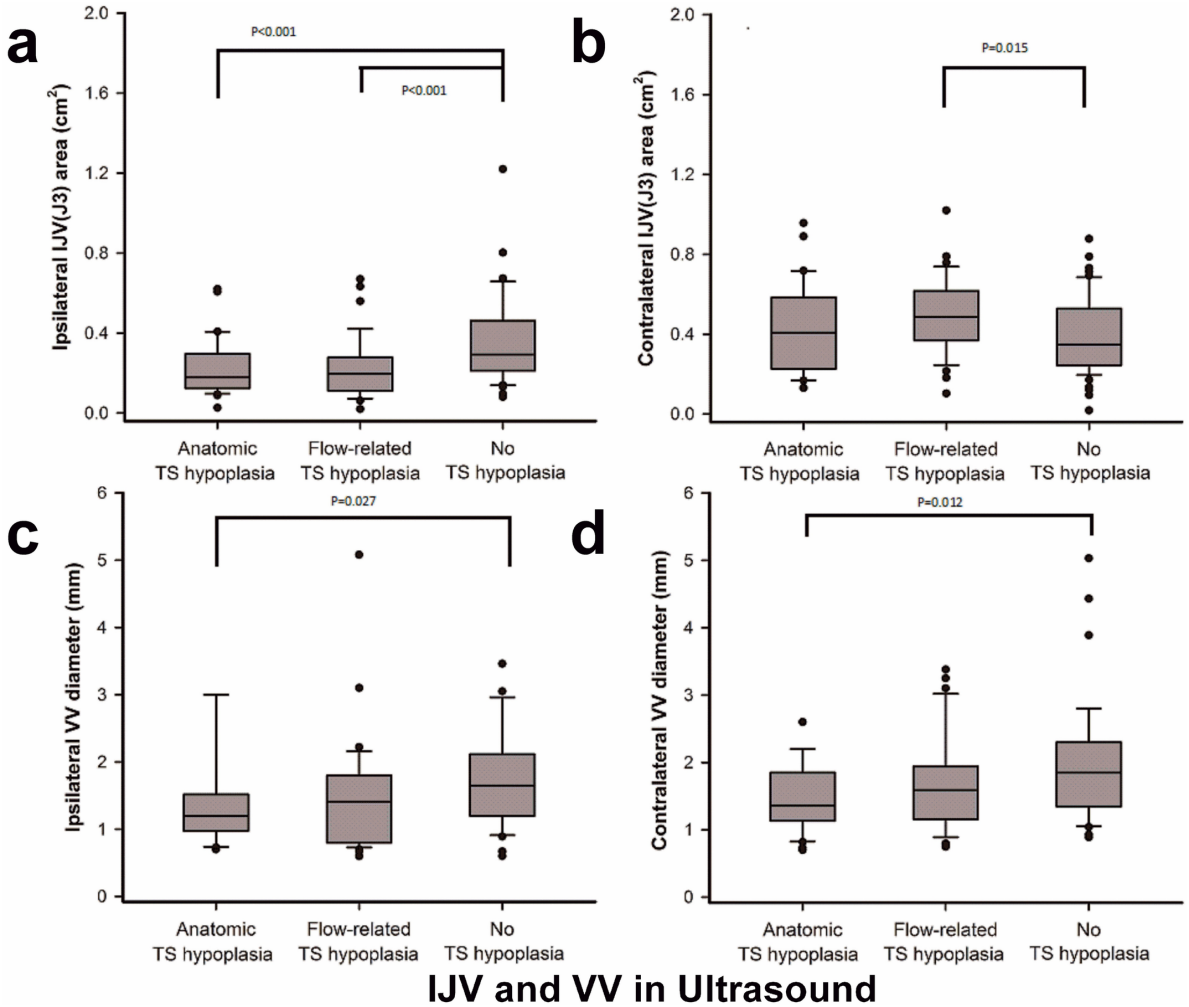
Between-group comparisons of the bilateral CSA of the J3 of the IJV/diameter of the VV. The CSAs (cm2) of ipsilateral J3 (A) and contralateral J3 (B) and the diameters (mm) of the ipsilateral vertebral vein (VV) (C) and contralateral VV (D) were measured by ultrasound. Data are presented as the median (interquartile range, IQR), and 80% of study subjects are represented between the upper and lower bars.

Recordings were made during brief apnea after three respiratory statuses: (1) normal respiration (resting or baseline) (Fig 1A and 1D), (2) deep inspiration (Fig 1B and 1E), and (3) expiration (Fig 1C and 1F). Subjects were asked not to strain during apnea to prevent increases in intra-thoracic pressure. The time-average-mean velocity (TAMV, cm/s) and cross-sectional lumen area (CSA, cm^2^) were recorded from the middle segment (J2) and upper segment (J3) of the IJV [16]. For TAMV acquisition, the Doppler cursor was positioned parallel to the vessel and the gate was adjusted to include the entire lumen. TAMV measurements were made using built-in software (iU22; Philips) and included at least three cardiac cycles on the Doppler spectrum. The probe was then rotated 90° to measure the CSA. The CSA was measured three times in B-mode and averaged for later analysis. Flow volume (FV, ml/min) was defined as equal to TAMV × CSA.

Since it is difficult to directly measure the CSA of the vertebral vein (VV), we recorded the diameter and TAMV from the V2 segment of the VV and defined the CSA as equal to π × r^2^, where r = VV diameter/2.

All sonography data were reviewed by two trained neurologists who were blinded to the clinical characteristics of subjects. The inter-rater reliability coefficient was greater than 0.90 for all IJV parameters.

### Statistical analysis

Size measurements of the TS, IJV, and VV are presented as the median and inter-quartile range (IQR). Continuous data were compared using the nonparametric Kruskal-Wallis test, and post hoc pairwise comparisons were conducted using the Wilcoxon rank-sum test with the Bonferroni correction. The relationships between TS hypoplasia and each ultrasound parameter (i.e., CSA and FV at each segment of the bilateral IJVs and VVs; side-to-side CSA differences, and side-to-side FV ratios) were plotted. The ability of ultrasound parameters to distinguish TS hypoplasia was examined using receiver-operating characteristic (ROC) curve analyses. The discriminating power of diagnosis is expressed in terms of the area under the ROC curve, ranging from 0.5 (no discrimination) to 1.0 (perfect discrimination). Two-sided *P* values < 0.05 were considered to indicate statistical significance. All analyses were performed with SAS version 9.2 (SAS Institute, Cary, NC, USA).

## Results

A total of 131 subjects (90 patients and 41 controls; 70 men and 61 women; mean age, 54.0 ± 15.1 years; age range, 17–86 years) were enrolled in the present study between the years of 2008 and 2012. The detailed distribution of subjects with and without TS hypoplasia was reported in Table 1 of our previous paper [10]. In summary, 38% (17), 33% (8), 43% (9) and 68% (28) of the subjects with TGA, TMB, panic disorder, and controls, respectively, are with no TS hypoplasia.

**Table 1:** Ultrasound parameters for the internal jugular veins and vertebral veins in subjects with and without transverse sinus hypoplasia.

Side	Vein	Respiratory status	Variables	TS hypoplasia Yes (n = 69)	TS hypoplasia No (n = 62)	*P* value
Ipsi	J3	Rest	TAMV	9.47 (3.31–17.2)	10.5 (7.63–14.2)	0.433
		Rest	CSA	0.19 (0.12–0.28)	0.29 (0.21–0.46)	< 0.001
		Rest	FV	113.8 (32.0–204.2)	226.8 (109.4–408.4)	< 0.001
	VV	Rest	TAMV	2.71 (1.00–5.20)	3.50 (2.41–5.58)	0.046
		Rest	Diameter	1.28 (0.90–1.80)	1.65 (1.20–2.10)	0.019
		Rest	FV	2.65 (0.47–6.50)	4.79 (1.61–9.96)	0.021
Contra	J3	Rest	TAMV	18.6 (14.1–23.3)	15.25 (10.4–19.6)	0.003
		Rest	CSA	0.41 (0.29–0.58)	0.35 (0.25–0.53)	0.066
		Rest	FV	450.5 (263.6–668.7)	367.5 (165.5–465.0)	0.001
	VV	Rest	TAMV	8.38 (4.38–11.3)	5.00 (3.00–7.05)	0.001
		Rest	Diameter	1.53 (1.16–1.91)	1.85 (1.35–2.30)	0.017
		Rest	FV	7.69 (3.28–15.0)	7.03 (2.39–16.21)	0.837

Data are presented as the median (interquartile range, IQR)

MR imaging findings have been previously published [10]. Briefly, of 131 included subjects, 69 subjects were diagnosed with TS hypoplasia according to MRV criteria, 62 with no hypoplasia. Of these 69 subjects, 30 were with anatomical TS hypoplasia and 39 were with flow-related TS hypoplasia due to downstream venous compression/stenosis of the IJV or left BCV.

A summary of ultrasound parameters in all subjects is presented in Table 1. In the subjects without TS hypoplasia, the smaller IJV were considered the ipsilateral side and were used for comparison with the IJV in subjects with TS hypoplasia. The median CSA (cm^2^) of the upper segment (J3) of the ipisilateral IJV was significantly smaller in subjects with TS hypoplasia than in subjects without TS hypoplasia (0.19 [0.12–0.28] vs. 0.29 [0.21–0.46], *P* < 0.001) (Table 1), and a post hoc analysis showed that the median CSA (cm^2^) was comparable between subtypes of TS hypoplasia, but significantly smaller in anatomical TS hypoplasia (0.18 [0.13–0.30] vs. 0.29 [0.21–0.46], *P* < 0.001) and flow-related TS hypoplasia (0.20 [0.11–0.28] vs. 0.29 [0.21–0.46], *P* < 0.001) subjects than in subjects without TS hypoplasia (Fig 2A; Table 2). Ipisilateral TAMV values were not statistically different between TS hypoplasia subjects and subjects without TS hypoplasia (9.47 [3.31–17.2] vs. 10.5 [7.63–14.2], *P* = 0.433) (Table 1).

**Table 2:** Ultrasound parameters of the internal jugular veins and vertebral veins at rest.

Side	Vein	Variables	Anatomical TS hypoplasia (n = 30)	Flow-related TS hypoplasia (n = 39)	No TS hypoplasia (n = 62)	P value
Ipsi	J3	TAMV	9.06 (5.60–17.2)	9.91 (2.64–17.0)	10.5 (7.63–14.2)	0.732
		CSA	0.18 (0.13–0.30)^1^	0.20 (0.11–0.28)^2^	0.29 (0.21–0.46)	<0.001
		FV	125.9 (47.3–192.7)^1^	100.4 (23.2–232.5)^2^	226.8 (109.4–408.4)	<0.001
	VV	TAMV	3.23 (1.48–5.60)	2.45 (1.00–4.52)^3^	3.50 (2.41–5.58)	0.036
		diameter	1.20 (1.00–1.50)^4^	1.41 (0.80–1.80)	1.65 (1.20–2.10)	0.067
		FV	2.63 (1.06–5.69)	2.65 (0.30–7.26)	4.79 (1.61–9.96)	0.055
Contra	J3	TAMV	19.5 (13.5–29.0)^5^	17.0 (14.1–22.2)^6^	15.25 (10.4–19.6)	0.008
		CSA	0.41 (0.23–0.57)	0.49 (0.36–0.62)^7^	0.35 (0.25–0.53)	0.042
		FV	470.0 (242.0–679.1)	450.5 (325.4–735.7)^8^	367.5 (165.5–465.0)	0.003
	VV	TAMV	8.73 (6.00–13.2)^9^	6.80 (3.81–10.2)	5.00 (3.00–7.05)	0.002
		diameter	1.36 (1.14–1.80)^10^	1.59 (1.16–1.94)	1.85 (1.35–2.30)	0.048
		FV	10.43 (3.81–15.97)	6.58 (2.69–17.29)	7.03 (2.39–16.21)	0.899
Bil	J3	FV	608.2 (352.7–775.3)	634.0 (459.1–836.7)	611.0 (383.8–777.7)	0.469
	VV	FV	11.71 (5.60–21.55)	8.60 (6.31–24.2)	12.24 (6.97–23.93)	0.606

Data are presented as the median (interquartile range, IQR). In post hoc analyses:

^1^: Anatomical TS hypoplasia vs. No TS hypoplasia, *P* < 0.001,

^2^: Flow-related TS hypoplasia vs. No TS hypoplasia, *P* < 0.001,

^3^: Flow-related TS hypoplasia vs. No TS hypoplasia, *P* = 0.011,

^4^: Anatomical TS hypoplasia vs. No TS hypoplasia, *P* = 0.027,

^5^: Anatomical TS hypoplasia vs. No TS hypoplasia, *P* = 0.004,

^6^: Flow-related TS hypoplasia vs. No TS hypoplasia, *P = 0*.*034*,

^7^: Flow-related TS hypoplasia vs. No TS hypoplasia, *P = 0*.*015*,

^8^: Flow-related TS hypoplasia vs. No TS hypoplasia, *P = 0*.*001*,

^9^: Anatomical TS hypoplasia vs. No TS hypoplasia, *P* = <0.001

^10^: Anatomical TS hypoplasia vs. No TS hypoplasia, *P = 0*.*012*

Contralateral CSA values were not statistically different between TS hypoplasia subjects and subjects without TS hypoplasia (0.41 [0.29–0.58] vs. 0.35 [0.25–0.53], *P* = 0.066) (Table 1), but a post hoc analysis showed that contralateral CSA was significantly larger in the subgroup with flow-related TS hypoplasia than in the subjects without TS hypoplasia (0.49 [0.36–0.62] vs. 0.35 [0.25–0.53], *P* = 0.015) (Fig 2B). TAMV values in the contralateral J3 were significantly greater in subjects with anatomical TS hypoplasia (19.5 [13.5–29.0] vs. 15.25 [10.4–19.6], *P* = 0.004) and subjects with flow-related TS hypoplasia (17.0 [14.1–22.2) vs. 15.25 [10.4–19.6], *P* = 0.034) than in subjects without TS hypoplasia, but were comparable between the subtypes of TS hypoplasia (Table 2). With regard to contralateral-ipsilateral CSA and FV differences, the bilateral IJVs appeared to be more asymmetrical in subjects with TS hypoplasia than in subjects without TS hypoplasia.

The ultrasound findings of diameter and flow profiles at the V2 segment of the vertebral vein revealed that the ipsilateral VV diameter in subjects with anatomical TS hypoplasia was significantly smaller than the corresponding VV diameter in subjects without TS hypoplalsia (1.36 [1.14–1.80] vs. 1.85 [1.35–2.30], *P* = 0.012) (Fig 2D), but the contralateral VV diameter was not significantly different between groups (1.20 [1.00–1.50] vs. 1.65 [1.20–2.10], *P* = 0.027) (Fig 2C). The total FV (the sum of FVs drained from the bilateral J3s and VVs) was not significantly different between groups (Table 2).

Using resting flow profiles, the ability of IJV and VV ultrasound parameters to discriminate TS hypoplasia diagnosed by MRV was analyzed by means of the ROC curve method (Table 3). Areas under the curve for the CSA and FV of ipsilateral J3 during rest, the CSA of ipsilateral J3 during inspiration and expiration, the FV of ipsilateral J2 during rest, the CSA of ipsilateral J2 during inspiration, the FV_J3_ ratio (FV of contralateral J3/FV of ipsilateral J3) during rest, and the CSA_J3_ ratio (CSA of contralateral J3/CSA of ipsilateral J3) during rest were all > 0.70 and statistically significant with 95% confidence. The CSA_J3_ ratio (contralateral-ipsilateral difference in CSA at J3) during rest had the most power for discriminating TS hypoplasia (area under the curve = 0.7924). The cut-off point that provided the best compromise between the sensitivity and specificity of the CSA_J3_ ratio during rest was > 1.55, which had a sensitivity of 0.80, a specificity of 0.81, and a positive predictive value of 0.82 (Table 4).

**Table 3:** Ultrasound parameters for distinguishing transverse sinus (TS) hypoplasia determined by receiver-operating characteristic (ROC) curve analyses.

side	vein	respiratory status	Variables	Area	SE	95% Wald Confidence limits	
Ipsi	J3	Rest	CSA	0.7412	0.0428	0.6573	0.8252
	J3	Rest	FV	0.7083	0.0452	0.6197	0.7968
	J3	Inspiration	CSA	0.7524	0.0425	0.6692	0.8357
	J3	Inspiration	FV	0.7553	0.0422	0.6726	0.8380
	J3	Expiration	CSA	0.7529	0.0429	0.6708	0.8368
	J2	Rest	FV	0.7354	0.0445	0.6481	0.8227
	J2	Inspiration	CSA	0.7377	0.0432	0.6531	0.8223
Ratio (contra/ipsi)	J3	Rest	CSA	0.7924	0.0411	0.7119	0.8730
	J3	Rest	FV	0.7389	0.0436	0.6535	0.8247

All 95% confidence limits did not include 0.5 and were thus statistically significant.

**Table 4:** The sensitivity, specificity, and positive predictive values (PPVs) for various cut-off points in the cross-sectional lumen area (CSA) ratio of the upper segment of internal jugular vein (J3).

	Ratio of CSA_J3_ during Rest		
Cut-off point	1.20	1.55	2.00
Sensitivity	0.86	0.80	0.57
Specificity	0.55	0.81	0.87
PPV	0.68	0.82	0.83

## Discussion

### Size of the TS on MR imaging is predicted by size of the IJV on ultrasound

A main finding was that ultrasound of the jugular vein provided reliable detection of TS hypoplasia as diagnosed with MRV. To this end, a cut-off CSA_J3_ ratio (CSA of contralateral J3 to TS hypoplasia /CSA of ipsilateral J3 to TS hypoplasia) of > 1.55 showed good sensitivity, specificity, and positive predictive value for discriminating TS hypoplasia. These findings are consistent with the results of a previous autopsy study indicating that the size of the TS predicts the size of the IJV [11]. Furthermore, these data suggest that ultrasound of the jugular veins is a potential complementary or surrogate diagnostic tool for the study of TS hypoplasia. Moreover, compared with the morphological changes of MR imaging, ultrasound is more sensitive to hemodynamic changes in the jugular vein, such as the cross-sectional area, blood flow velocity, and blood flow. It can also be examined in different body positions, such as supine and standing positions, and respiratory conditions, such as at rest, inspiration, expiration and the Valsalva maneuver, which is consistent with the physiological characteristics of the veins. These are the unique advantages of ultrasound relative to MR imaging. In addition, ultrasound is advantageous because it is non-invasive, inexpensive, repeatable, and easy to perform at the bedside. In the study, we only analysed the data of the distal segment (J3) of the IJV, and did not use the data of proximal segment (J1) or middle segment (J2) of the IJV. Because the distal segment (J3) of the IJV originates from the sigmoid sinus and via superior jugular bulb, draining the venous flow almost only from TS. However, venous flow in the J2 or J1 receives the flow from some venous collaterals, and especially in the case of TS hypoplasia, a considerable portion of venous flow in J1 and J2 is not from TS. Therefore, there was no reason to use the venous flow in J1 or J2 to predict the flow from the TS. Thus, we did not present the data in J1 and J2.

### Clinical implications of a small TS and small IJV with compensatory high flow velocity and volume

Many previous reports have detailed the adverse hemodynamic effects of TS asymmetry [4–7,19]. A number of studies have also described other clinical implications: Afanas’eva et al. reported that IJV asymmetry correlates with specific variations and variabilities of blood pressure in patients [20]. Additionally, Krsmanović et al. reported that small IJVs with restricted flow are independently associated with disease severity in multiple sclerosis patients [21]. Given our observation of the relationship between small IJV diameter and TS hypoplasia, the above-mentioned adverse associations are not surprising.

### The IJV lumen contralateral to TS hypoplasia is not subject to compensatory enlargement

In subjects with anatomical TS hypoplasia, the diameters of the bilateral VVs and contralateral IJV did not demonstrate compensatory enlargement on ultrasound (Fig 2); however, flow velocities and volumes were increased. These ultrasound findings are consistent with those of our MR imaging study, in which the contralateral TS diameter did not demonstrate compensatory enlargement on contrast T1, but compensatory changes in flow were observed with MRV [10]. These data discredit the common belief that compensatory dilatation occurs contralateral to TS hypoplasia, and instead suggest that compensatory changes occur in flow velocity and volume.

### Clinical implications of compensatory increases in IJV flow velocity and volume contralateral to TS hypoplasia

We observed that while the ipsilateral IJV and VV were anatomically smaller, the contralateral IJV and VV were not subject to compensatory enlargement in patients with anatomical TS hypoplasia. However, flow velocities and volumes were increased in the contralateral IJV and VV, and the total amount of venous drainage was comparable in all subjects (Table 2). According to the Poiseuille's law, higher intracranial venous pressure is needed to achieve compensatory TAMV to maintain adequate cerebral venous drainage in smaller veins, such that blood flow should move faster in the bilateral VVs and contralateral IJV. Consistent with this principle, we observed increased flow volume and flow velocity in the bilateral VVs and contralateral IJV in patients with TS hypoplasia (Table 2). Of note, the hemodynamic impact of high flow velocity and volume on smaller veins requires additional study. Previous work by our group revealed that flow-related TS hypoplasia is more highly associated with clinical neurological disorders such as TGA and TMB, in which venous pathogenesis may be related to the transmission of venous pressure [10]. The possibility of upstream intracranial venous hypertension in subjects with anatomical TS hypoplasia warrants further clinical investigation.

### Outlook

Though the present study validates the utility of ultrasound for detecting TS hypoplasia, it was difficult to distinguish anatomical TS hypoplasia from flow-related TS hypoplasia using the resting or baseline CSA size and the FV of the IJV. For the time being, it seems that ultrasound with high-resolution B scan flow parameters cannot replace MR imaging for distinguishing types of TS asymmetry. However, the analysis other ultrasound parameters may provide future utility for this endeavor; other parameters have been used to detect significant differences in flow volume between IJVs with and without venous compression at various segments, the emergence of echo contrast proximal to sites of venous compression, acceptable ultrasound parameter cut-off points for predicting compression of the brachiocephalic vein, and the absence of reflow appearance during the Valsalva maneuver in cases of IJV compression [22]. Additionally, specific hemodynamic information related to the IJV has been obtained through the use of respiratory maneuver, such as paradoxical IJV flow in response to deep inspiration [12]. In future studies, we hope to employ these additional parameters to reliably and clearly distinguish anatomical TS hypoplasia from flow-related TS hypoplasia using ultrasound.

### Limitations

This study has limitations. First, the sample size is small, and there is lack of a standard protocol and reference ranges for ultrasound diameter in the literature. However, in our ultrasound laboratory, we have more than 10 years of experience in the venous ultrasound examination and have published articles introducing our jugular venous study protocol and the results of jugular venous ultrasound data in various diseases [8,9,12,15,16]. Since there is no standard reference ranges with large sample size in the literatures, in this study, we did our best to include the ultrasound data of both patients and controls. We hope that more studies will confirm our diagnostic criteria, and we will continue to collect study subjects and expand the sample size to further verify and improve the accuracy of the diagnostic criteria of TS hypoplasia. Second, while we purport the accuracy of our conclusions and soundness of our methods, the following limitations should be considered. Our study population had a high prevalence of venous outflow obstruction. The frequency of TS hypoplasia may be different in populations without venous outflow obstruction; however, because the purpose of this study was to compare differences between the ultrasound parameters of the IJVs and VVs in subjects with and without TS hypoplasia, the results are unlikely to be influenced by the prevalence of TS hypoplasia in the study population. Third, although we prospectively collected data from MR imaging and the ultrasound studies, the data were reviewed retrospectively. This study may therefore be subject to the bias of a retrospective study.

## Conclusions

In conclusion, IJV flow profiles on ultrasound appear to provide reliable detection of TS hypoplasia, but future work is required to optimize this imaging modality for the differentiation of TS hypoplasia subtypes. Additionally, we observed indicators of upstream intracranial venous hypertension in subjects with anatomical TS hypoplasia. Future work is needed to clarify the clinical implications of each subtype of TS hypoplasia.

## Supporting information

S1 Table This is the S1 Table title. The parameters of ultrasound jugular venous flow profiles in study subjects with and without transverse sinus hypoplasia.
(XLS)
